# Initial Evaluation of Prospective and Parallel Assessments of Cystic Fibrosis Newborn Screening Protocols in Eastern Andalusia: IRT/IRT versus IRT/PAP/IRT

**DOI:** 10.3390/ijns5030032

**Published:** 2019-09-03

**Authors:** Ilham Sadik, Inmaculada Pérez de Algaba, Rocío Jiménez, Carmen Benito, Javier Blasco-Alonso, Pilar Caro, Víctor M. Navas-López, Javier Pérez-Frías, Estela Pérez, Juliana Serrano, Raquel Yahyaoui

**Affiliations:** 1 Clinical Laboratory, Hospital La Línea de la Concepción, 11300 Cádiz, Spain; 2 Laboratory of Metabolic Disorders and Newborn Screening Center of Eastern Andalusia, Málaga Regional University Hospital, Avenida Arroyo de los Angeles s/n, 29011 Málaga, Spain; 3 Department of Genetics, Málaga Regional University Hospital, 29011 Málaga, Spain; 4 Department of Pediatrics, Málaga Regional University Hospital, 29011 Málaga, Spain; 5 Institute of Biomedical Research in Málaga-IBIMA, 29010 Málaga, Spain; 6 Department of Pharmacology and Pediatrics, University of Málaga, 29071 Málaga, Spain

**Keywords:** cystic fibrosis, newborn screening (NBS), dried blood spot (DBS), immunoreactive trypsinogen (IRT), pancreatitis-associated protein (PAP)

## Abstract

Identifying newborns at risk for cystic fibrosis (CF) by newborn screening (NBS) using dried blood spot (DBS) specimens provides an opportunity for presymptomatic detection. All NBS strategies for CF begin with measuring immunoreactive trypsinogen (IRT). Pancreatitis-associated protein (PAP) has been suggested as second-tier testing. The main objective of this study was to evaluate the analytical performance of an IRT/PAP/IRT strategy versus the current IRT/IRT strategy over a two-year pilot study including 68,502 newborns. The design of the study, carried out in a prospective and parallel manner, allowed us to compare four different CF-NBS protocols after performing a post hoc analysis. The best PAP cutoff point and the potential sources of PAP false positive results in our non-CF newborn population were also studied. 14 CF newborns were detected, resulting in an overall CF prevalence of 1/4, 893 newborns. The IRT/IRT algorithm detected all CF cases, but the IRT/PAP/IRT algorithm failed to detect one case of CF. The IRT/PAP/IRT with an IRT-dependent safety net protocol was a good alternative to improve sensitivity to 100%. The IRT × PAP/IRT strategy clearly performed better, with a sensitivity of 100% and a positive predictive value (PPV) of 39%. Our calculated optimal cutoffs were 2.31 µg/L for PAP and 167.4 µg^2^/L^2^ for IRT × PAP. PAP levels were higher in females and newborns with low birth weight. PAP false positive results were found mainly in newborns with conditions such as prematurity, sepsis, and hypoxic-ischemic encephalopathy.

## 1. Introduction

Cystic fibrosis (CF) is a common autosomal recessive disorder that affects the sweat glands and the digestive, respiratory, and reproductive systems due to genetic variants in the cystic fibrosis transmembrane conductance regulator (*CFTR*) gene [1]. These variants result in defective chloride transport at the apical surface of epithelial cells [2]. More than 2000 variants of the *CFTR* gene have been reported [3]. Some of these variants are disease causing, some do not cause CF, some are associated with single or milder organ system involvement, and some are of uncertain clinical significance [3,4]. Among CF-affected children, 85 to 90% develop a life-threatening chronic disease with pancreatic exocrine insufficiency and respiratory tract abnormalities that are associated with recurrent bronchopulmonary infections [5,6].

Identifying newborns at risk for CF by newborn screening (NBS) using dried blood spot (DBS) specimens provides an opportunity for presymptomatic detection before irreversible pathologies develop [7,8]. NBS for CF is performed in a growing number of countries around the world. There is broad agreement on the benefits of NBS for CF in terms of reducing morbidity and mortality, thus reducing the burden on the healthcare system and costs [9,10]. Many detection programs have been developed in Europe since the end of the 1980s, though there is marked variation in the designs of the NBS protocol [11]. However, given the considerable geographical, ethnic, and economic variations, a single approach would not be appropriate [12,13]. 

All NBS strategies for CF begin with measuring immunoreactive trypsinogen (IRT) in DBS specimens [14]. IRT is a nonspecific marker that increases in most CF patients. It may also increase for other reasons, including prematurity and low birth weight, resulting in an elevated rate of false positives and anxiety for the families [15,16]. For this reason, additional tiers are required to improve the specificity of the screening [17,18]. The two most commonly used algorithms are IRT/IRT (analyzing the concentration of IRT in a second DBS specimen collected between 10–28 days of life, when the marker has greater specificity) and IRT/DNA (analyzing a panel of *CFTR* pathogenic variants in the initial DBS specimen) [11,12]. 

The IRT/DNA protocol is probably the most widely used strategy and has the highest level of sensitivity, which provides good specificity [11,19]. However, using DNA analysis as part of a screening procedure leads to unwanted detection of CF carriers and inconclusive diagnoses [20,21]. Another limitation is that it does not offer equal coverage for all ethnic groups, so this strategy has certain legislative and ethical implications that should be assessed when including it into a neonatal screening program [22,23]. 

Programs that use the IRT/IRT algorithm avoid the issue of carrier detection. IRT concentrations typically decrease as the newborn ages, so NBS programs using this strategy usually calculate their IRT cutoffs for age ranges that includes the early newborn period (1–7 days of life) and the later newborn period (10–28 days of life) [24]. CF-NBS programs may use a fixed or floating IRT cutoff concentration, depending on if they have consistent or variable cutoff for the specimens tested [11]. The drawbacks of this approach are that a second specimen must be collected, there is lower sensitivity and specificity, and there is a possibility of missed cases or delayed diagnosis [25,26]. 

In the past decade, an alternative, purely biochemical protocol has emerged. This protocol involves measuring pancreatitis-associated protein (PAP) as second-tier testing [27]. PAP is a secretory protein produced by the pancreas under stress conditions. It is more specific than IRT for CF-NBS [28]. Some pilot studies have evaluated the use of PAP in different CF-NBS protocols and found that introducing PAP screening reduces the number of infants identified as carriers and the number of inconclusive diagnoses. Furthermore, it may reduce costs [9,17,29,30,31,32,33]. Nevertheless, the information available on its analytical performance in CF-NBS is still very scarce due to the fact that its use has been limited to some European NBS centers in Germany, France, Portugal, the Netherlands, and Spain.

In Spain, there are currently 19 neonatal screening programs. A standard panel that includes screening for 7 diseases-including CF-has been mandatory since 2015 [34]. Most programs had been screening for CF previously, but there was no standard strategy. In Andalusia, CF-NBS using the IRT/IRT algorithm followed by a quantitative sweat chloride test (SCT) was introduced in 2011. However, this strategy has yielded an unacceptable false positive rate (0.23%) and generated a lot of stress for the families [16] and for these reasons we considered evaluating the introduction of PAP measurement as a second-tier testing. 

The objective of this work was to evaluate the analytical performance of an IRT/PAP/IRT strategy versus the current IRT/IRT strategy in our center over a two-year pilot study. The aim was to evaluate whether making PAP measurement in CF-NBS the standard in our region would be more suitable than the current screening algorithm. Moreover, we attempted to identify the optimal test combinations and best cutoff points for predicting CF. A secondary aim was to compare our cutoff points to other published recommendations and study the potential sources of PAP false positive results in our non-CF newborn population.

## 2. Material and Methods

### 2.1. Study Design and Cohort

This project was approved by the Ethics Committee of the Province of Málaga, Spain, in January 2017 and was conducted at the NBS Center of Eastern Andalusia located in the Málaga Regional University Hospital. The pilot study for CF-NBS started at June 2017 and data were collected from newborns born between June 2017 and May 2019. This study has been carried out in a prospective and parallel manner so that the analytical performance parameters of both algorithms could be compared simultaneously.

The prospective study included all 68,502 newborns screened for CF in the NBS program. DBS specimens for CF screening were collected at maternity hospitals and local health centers from all over Eastern Andalusia. The recommended blood collection time was from 48 to 72 h after birth, though some samples were collected later, at 6–7 days of life. NBS collection devices were sent to our laboratory mainly by postal mail. Unsatisfactory DBS specimens were rejected. For those cases, a second DBS specimen was requested and collected in approximately the 2nd week of life. 

### 2.2. IRT and PAP Measurements and Screening Protocols

**Figure 1:**
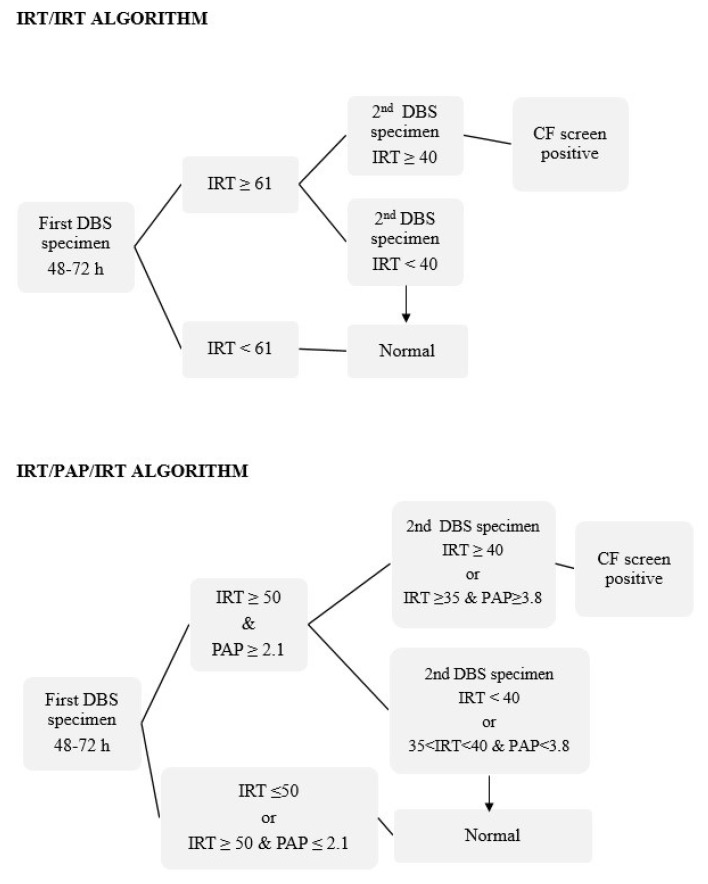
Algorithms used in parallel during the pilot study for cystic fibrosis newborn screening (CF-NBS) in Eastern Andalusia. Immunoreactive trypsinogen (IRT) is expressed in ng/mL and pancreatitis-associated protein (PAP) is expressed in μg/L. PAP concentration was routinely measured in the initial DBS specimen.

IRT quantification was performed via a time-resolved immunofluorometric assay, using the AutoDELFIA^®^ autoanalyzer (PerkinElmer, Turku, Finland). PAP quantification was performed using a sandwich time-resolved immunofluorometric assay, using the MucoPAP-F kit (Dynabio, Marseille, France) and in accordance with the manufacturer’s instructions. The IRT/IRT and IRT/PAP/IRT strategies were carried out in parallel, as shown in Figure 1. 

In the IRT/IRT protocol, the IRT cutoff value for the initial DBS specimen was set at 61 ng/mL. This fixed cutoff point was based on the 99th percentile of our first 10,000 newborns in the study population. This cutoff was validated annually. If IRT values were above 50 ng/mL, the IRT screening was repeated twice more on the first sample. If any of the three measurements was ≥61 ng/mL, a second DBS specimen was requested for IRT measurement. The second DBS specimens were typically collected in the 4th week, when the newborn was between 24 and 28 days of life. The IRT cutoff value for the second specimen was set at 40 ng/mL. Infants who had IRT ≥40 ng/mL were considered as CF screen positives and referred to our specialized CF clinical center for a clinical evaluation and SCT.

In the IRT/PAP/IRT protocol, an IRT value ≤ 50 ng/mL or an IRT value ≥ 50 ng/mL and a PAP value ≤ 2.1 µg/L were considered normal. If the initial specimen had an IRT value ≥ 50 ng/mL and a PAP value ≥ 2.1 µg/L, a second specimen was requested. The lower cutoff limit of 50 ng/mL was set in this approach in order to evaluate whether including a PAP measurement would detect potential CF true positives cases which had IRT concentrations of between 50 and 61 ng/mL. In regards to the PAP value, the provisional cutoff points were set in accordance with the available bibliography. For the second DBS specimen, the cutoff value for a CF positive screen was either IRT ≥ 40 ng/mL or an IRT value between 35 and 40 ng/mL and PAP ≥ 3.8 µg/L. A normal screen was thus defined as either IRT < 40 ng/mL or IRT between 35 and 40 ng/mL and PAP < 3.8 µg/L on the second specimen. PAP concentration was routinely measured in the initial DBS specimen. If PAP determination was not possible in the initial DBS specimen, it was measured along with IRT in the second DBS specimen.

### 2.3. CF Diagnosis

CF diagnosis was defined as a sweat chloride concentration ≥60 mmol/L and/or detection of two pathogenic variants of the *CFTR* gene. SCT was performed according to a standardized protocol via quantitative pilocarpine iontophoresis with the Macroduct^®^ collection method. Sweat chloride concentration was measured in a Model 926S MK II Chloride Analyzer (Sherwood Scientific Ltd., Cambridge, UK). Genetic studies in peripheral blood were performed using an Elucigene^®^ CF-EU2v1 50-mutation kit (Elucigene Diagnostics, Manchester, UK) and an Elucigene^®^ CF Iberian Panel kit (screening for 12 mutations that are prevalent in the Iberian Peninsula). For cases with positive SCT and none or only one CF mutation identified, complete sequencing of the *CFTR* gene using next-generation sequencing was performed. Patients’ parents were counseled on and consented to genetic testing as part of the patients’ clinical diagnosis. CF-false negatives cases are communicated to our NBS laboratory by our regional CF clinical center.

### 2.4. PAP False Positive Values

To evaluate the potential causes of PAP false positive values, demographic and clinical data from the electronic medical records of newborns with PAP concentration above 3.8 µg/L were collected and reviewed.

### 2.5. Discrimination Analysis and Protocol Comparison

Data were collected in a Microsoft Access 2017 database and in our NBS program database. Statistical analysis of the protocols’ performance was carried out using the IBM SPSS Statistics 20.0 software package for Windows. PAP data were expressed as medians and qualitative variables as percentages. Non-CF newborns whose IRT and/or PAP concentrations were outliers from the expected distribution were not included in the statistical analysis. The hypothesis was tested using the Student’s *t* test for unpaired samples. A linear regression model was used to evaluate the influence of each specific variable on PAP concentration. In all cases, the null hypothesis rejection value was defined as α ≤ 0.05. 

Discriminant analysis for second-tier testing of all screen-positive cases was performed by determining areas under curve (AUCs). AUCs were calculated for logarithmic concentrations of IRT and PAP both separately and combined as a product, as suggested by Weidler S. et al. [35]. To identify the optimal PAP and IRTxPAP cutoff values, sensitivity and (1-specificity) were calculated for each lg value.

Analytical performance metrics for both protocols (sensitivity, specificity, positive predictive value (PPV), negative predictive value (NPV), and their 95% confidence intervals) were calculated and compared. Given the results found and the fact that we had data available, we were also able to analyze two other protocols: an algorithm consisting of the IRT/PAP/IRT protocol with an IRT-dependent safety net (SN) when the initial IRT value was ≥150 ng/mL as well as an IRTxPAP/IRT algorithm.

## 3. Results

A total of 68,502 newborns were screened during this CF-NBS pilot study. There were 14 CF-true positive cases identified, resulting in an overall CF prevalence of 1/4893 newborns. The NBS results and the clinical data on the infants diagnosed with CF are presented in Table 1. No CF-false negatives cases were reported at our NBS center. None of the newborns with a positive screening test had an initial SCT with an inconclusive result.

### 3.1. Performance Metrics 

In the IRT/IRT algorithm, 613 newborns (0.89%) had increased IRT concentrations (≥61 ng/mL) and a second specimen was requested. Of these, 593 second specimens were received and 93 of them (0.13%) also had increased IRT values (≥40 ng/mL). They were referred for clinical evaluation and SCT. 

For the IRT/PAP/IRT algorithm, 1056 newborns (1.54%) had increased IRT concentrations (≥50 ng/dL), but only 241 (0.35%) had increased PAP levels (≥2.1 µg/L). A second specimen was requested for those cases and 33 of them (0.04%) had increased levels of IRT in the second specimen (≥40 ng/mL). These newborns were referred for diagnosis confirmation. There were 2 cases which had IRT ≥ 50 ng/mL and PAP ≥ 3.8 µg/L in the first specimen and an IRT concentration between 35 and 40 ng/mL in the second specimen. They were also referred.

The performance metrics of both algorithms are described in Table 2. The IRT/IRT algorithm detected all CF cases, but the IRT/PAP/IRT algorithm missed 1 case (P11) because the patient presented with a normal PAP concentration (see Table 1). Given that both protocols were performed in parallel, we had the opportunity to evaluate a third algorithm which consisted of the IRT/PAP/IRT protocol with an IRT-dependent SN when initial IRT ≥ 150 ng/mL as well as a fourth algorithm, which consisted of the IRT × PAP/IRT protocol with an IRT cutoff of 40 ng/mL for the second specimen. Data on how these protocols performed are also shown in Table 2.

### 3.2. Discrimination Analysis 

For all newborns with IRT ≥ 50 ng/mL, we conducted a discriminant analysis between two different second-tier protocols in order to assess their ability to predict CF: PAP value and the product of the initial IRT × PAP. The power of discrimination of the initial IRT alone was also calculated. The product of IRT × PAP had a higher AUC—0.949 (95% CI, 0.922 to 0.976)—than PAP (0.898; 95% CI, 0.811 to 0.985) and IRT (0.933; 95% CI, 0.896 to 0.969). The calculated optimal cutoffs for second-tier parameters were 2.31 µg/L for PAP (if an IRT-dependent SN is used) and 167.4 µg^2^/L^2^ for IRTxPAP.

### 3.3. PAP Results in Non-CF Newborns and Investigation of Sources of False Positives

The median value of PAP in the 1042 non-CF newborns with initial IRT ≥ 50 ng/mL was 1.25 µg/L. Of these, 228 (21.88%) had PAP concentration above the established cutoff of 2.1 µg/L. Among the 443 non-CF newborns with initial IRT concentration between 50 to 61 ng/mL, 80 (18.1%) had PAP levels above this cutoff.

Males were observed to have significantly lower PAP concentrations than females (1.46 vs. 1.70 µg/L; *p* = 0.04). Low BW newborns (<2500 g) had higher PAP concentrations than normal BW newborns (1.91 vs. 1.58 µg/L; *p* = 0.20) and prematurity (GA < 37 weeks) was also associated with a higher PAP concentration (1.73 vs. 1.60 µg/L; *p* = 0.47), but these differences were not statistically significant. The influence of each specific variable (sex, birth weight, and gestational age) was analyzed using a linear regression model; birth weight was the only explanatory variable of PAP concentration (*p* = 0.003). 

Finally, we investigated potential sources of PAP false positive results in non-CF newborns with PAP concentration above 3.8 µg/L. Of the 71 newborns who met these criteria, 5 passed away due to significant complications due to prematurity or dysmorphic syndrome; 6 suffered from hypoxic-ischemic encephalopathy; 10 were very preterm; 5 had sepsis, which in some cases was associated with prematurity; 3 suffered severe intestinal obstruction (1 of them was later diagnosed with Hirschsprung disease); 1 had gastric perforation; 2 had renal malformations; and 1 suffered from severe heart disease. The remaining 39 newborns had no clinical history of interest. 

## 4. Discussion

In this study, we demonstrate that introducing PAP as second-tier testing in a CF-NBS program is more sensitive and specific for CF detection and is a better screening strategy than the IRT/IRT protocol. To fully validate the impact of introducing this biomarker in our CF-NBS program, we decided to perform an IRT/PAP/IRT algorithm in parallel with the current IRT/IRT protocol. We also decreased the initial IRT cutoff point from 61 to 50 ng/mL in order to evaluate if not only specificity but also sensitivity could be improved. This study design allowed us to compare four different CF-NBS strategies. Of these four, the IRT × PAP/IRT protocol was found to have the best performance indicators.

All CF-NBS programs should aim to maximize the correct diagnosis of CF while minimizing the false positive rate, the detection of carriers, and inconclusive diagnoses. When designing a CF-NBS program, timely and appropriate processing of the results should be guaranteed so as to minimize potential stress for families caused by *CFTR* mutation analysis or the need to collect a second DBS specimen [12]. Our experience using the IRT/IRT strategy over the last 8 years is that although we are able to detect CF newborns on time [12] and hardly ever detect carrier or cystic fibrosis screen positive with an inconclusive diagnosis (CFSPID) cases, our low PPV (15.1%), false positive rate (0.13%), and the number of second DBS specimens that must be requested are unacceptable. At the moment, genetic studies cannot be done in our region without written informed consent, so we were not able to evaluate introducing a *CFTR* mutation panel into the protocol in addition to measuring PAP concentration [13]. 

IRT quantification as the first step in CF-NBS does not guarantee 100% sensitivity [35]. In fact, our NBS program has had several cases of false negatives in recent years, especially in CF infants with pancreatic sufficiency [16]. We hypothesize that introducing PAP in the NBS algorithm could improve sensitivity if we decrease the initial IRT cutoff, but this premise could not be conclusively demonstrated in this study. PAP may also be a valuable tool for identifying CF newborns with meconium ileus whose IRT concentration may be normal [32]; we were not able to evaluate this hypothesis because we did not identify any cases with this clinical presentation during the study. 

Confirmatory diagnostic testing and a timely diagnosis after a positive screen result is essential [36]. The SCT remains the gold standard test for diagnosing CF [37]. The ideal timing for the SCT is before 4 weeks of age and when the newborn weighs more than 2000 g [38]. A sweat chloride concentration above 60 mmol/L is the definitive cutoff value for CF diagnosis. If a concentration between 30 and 59 mmol/L is found, the SCT should be repeated [38,39]. No CFSPID cases were identified during this CF-NBS pilot study. In cases where it was not possible to obtain a sufficiently large sweat sample in order analyze the chloride concentration (e.g., preterm newborns), conductivity was an indicative biochemical parameter, but a *CFTR* mutation analysis had to be performed (P8, P11, and P14).

In regards to the genetic characterization of our CF infants, the p.Phe508del was identified in 17 of the 28 *CFTR* variants found. A similar prevalence was reported in a previous study on a neonatal population in our region [16]. Five of the 28 *CFTR* variants were not identified by the *CFTR* Panel Mutation and we had to use EGA. Genetic analysis performed on P2 revealed one pathogenic variant (p.Leu206Trp) and one variant of uncertain significance (p.Arg285Gly). None of them were detected by the *CFTR* Panel Mutation, so this CF infant would have been missed in the event we had followed the IRT/DNA strategy. However, CF would have been able to be detected in P2 using PAP concentration, despite the fact the patient had pancreatic sufficiency. 

The results of this study support other authors’ findings that there is no ideal CF-NBS strategy. Given that PAP concentration failed to detect a CF newborn (P11), we believe that adjustments must be made to the IRT/PAP/IRT protocol. Other cases of PAP false negative results have been reported previously [29]. The IRT/DNA strategy would have missed one case (P2) and the IRT/PAP/DNA protocol would have missed one case (P11). Although this study was carried out on a small cohort with only 14 CF cases detected, our results suggests that an IRT/PAP/IRT + SN strategy could be a good alternative for optimizing sensitivity, as opposed to what Marcao A. et al. concluded in their study [32]. In this study, the IRT × PAP/IRT strategy, which was based on the study by Weidler, S. et al. [35], clearly performed better. It had a PPV of 39%, a very good value when compared to PPVs obtained using other purely biochemical CF-NBS algorithms described in the literature [30]. Surprisingly, our optimal IRT × PAP cutoff, calculated at 167.4 µg^2^/L^2^, is very similar to the cutoff of 165 µg^2^/L^2^ proposed by Weidler, S. et al. In addition, if we had used this strategy, the number of second DBS specimens requested would have been significantly lower: 181 compared to 613 with the IRT/IRT protocol, 241 with the IRT/PAP/IRT protocol, and 192 with the IRT/PAP/IRT + SN protocol. For all these reasons, we are evaluating implementation of this strategy in our CF-NBS program, although there is insufficient time to know whether there are additional CF-false negatives cases and the performance parameters may require recalculation in the future.

The last objective of this study was to evaluate the potential causes of PAP false positive results. PAP is synthesized in high amounts after pancreatic stress (e.g., pancreatitis, systemic infections, hypoxic events, and after abdominal surgery) [40,41]. Vernooij-van Langen et al. evaluated the potential effects of sex, gestational age, birth weight, blood transfusion, and time of heel prick on PAP blood concentrations in a newborn population in the Netherlands [41]. They concluded that low birth weight, blood transfusion, and collection time after seven days following birth may lead to significantly higher PAP concentrations. In our study, an association between higher levels of PAP and low birth weight was also observed. We found significantly lower PAP values in male newborns compared to female newborns. A similar trend was described by Vernooij-van Langen et al., but the difference was not statistically significant. We think that these results should be interpreted with caution. We do not recommend adjusting PAP cutoff values according to these variables unless these findings are corroborated by other studies. Finally, since it had not been previously studied in detail, we have investigated the potential clinical causes that may produce PAP false positive results. We found an association with prematurity, sepsis, hypoxic-ischemic encephalopathy, dysmorphic syndrome, intestinal abnormalities, and organ malformations, although surprisingly, more than half of the cases did not have any clinical presentation that could explain the high PAP concentration.

## 5. Conclusions

This is the first pilot study conducted in Spain that evaluates the introduction of the PAP concentration measurement in a CF-NBS program using the IRT/IRT protocol. The design of the study allowed for the evaluation of the analytical performance of four screening strategies (IRT/IRT, IRT/PAP/IRT, IRT/PAP/IRT + SN, and IRT × PAP/IRT). The IRT × PAP/IRT protocol had the best PPV and lowest number of second DBS specimens requested. This investigation has allowed us to make some adjustments to our protocol and to better know the causes that influence sensitivity and specificity of PAP in CF-NBS. 

## Figures and Tables

**Table 1:** NBS results and clinical data from CF infants identified during the CF-NBS pilot study.

Patient ID (Days Old at 1st and 2nd DBS Collection)	BW (g)	GA (Weeks)	1st IRT (ng/mL)	PAP (µg/L)	2nd IRT (ng/mL)	SCT (mmol/L)	Fecal Elastase (µg/g)	PI	*CFTR* Gene Pathogenic Variants	
P1 (3,24)	3400	40	115	5.23	41	73	464	N	p.Trp1282*	*c.3140-26A* > *G*
P2 (4,28)	2100	33	75	4.74	43	75	417	N	*p.Leu206Trp* ^ɸ^	*p.Arg285Gly* ^ɸ^
P3 (4,25)	2360	37	101	2.93	70	83	150	Y	*p.Phe508del*	*p.Phe508del*
P4 (3,24)	3400	40	110	2.32	90	101	24	Y	*p.Phe508del*	#
P5 (3,25)	3640	40	86	2.36	73	75	183	Y	*p.Phe508del*	*c.3140-26A* > *G*
P6 (3,25)	3350	40	114	5.13	80	90	<15	Y	*p.Phe508del*	*p.Phe508del*
P7 (3,24)	3190	39	77	4.49	98	85	128	Y	*p.Phe508del*	*p.Phe508del*
P8 (3,26)	3020	39	131	6.17	106	NA ^1^	<15	Y	*p.Phe508del*	*p.Phe508del*
P9 (6,28)	3345	37	154	>8.0	140	88	NA	Y	*p.Phe508del*	*p.Asn1303Lys*
P10 (3,24)	3000	38	153	7.64	150	94	<15	Y	*p.Phe508del*	*p.Ile507del*
P11 (4,25)	1340	29	170	0.99	71	NA	141	Y	*p.Phe508del*	*p.Gln685ThrfsX* ^ɸ^
P12 (3,26)	2700	38	155	7.40	207	87	<15	Y	*p.Gly673Ter* ^ɸ^	*p.Asn1303Lys*
P13 (5,25)	2890	39	180	6.69	226	95	<15	Y	*p.Phe508del*	*p.Phe508del*
P14 (4,19)	3200	37	137	>8.0	255	NA ^2^	<15	Y	*p.Phe508del*	*p.Phe508del*

BW: Birth weight. GA: gestational age. PI: pancreatic insufficiency. N: no. Y: yes. Fecal elastase (NV > 200 µg/g). # Complete CFTR gene sequencing is still in progress. NA: no result recorded. ^1^ Conductivity 120 mmol/L and ^2^ Conductivity 106 mmol/L (NV < 90). ^ɸ^ Missed by 50 + 12 CFTR Panel Mutation and identified by extended gene analysis (EGA).

**Table 2:** Performance of four different CF-NBS protocols for 68,502 newborns screened and 14 infants with CF detected during the pilot study.

Screening Protocol	IRT/IRT	IRT/PAP/IRT	IRT/PAP/IRT + SN	IRT × PAP/IRT
-CF-screen positives *(% of infants screened)*	93 (0.13%)	35 (0.05%)	66 (0.09%)	36 (0.05%)
-CF cases detected	14	13	14	14
-False positive cases *(% of positive test cases with appropriate follow-up)*	79 (0.11%)	22 (0.03%)	52 (0.07%)	22 (0.03%)
-False negative cases	0	1	0	0
-True negative cases	68.409	68.466	68.436	68.466
-Sensitivity (95% CI)	100% [96.4–100]	92.9% [75.7–100]	100% [96.4–100]	100% [96.4–100]
-Specificity (95% CI)	99.9% [99.8–99.9]	100% [99.9–100]	99.9% [99.9–100]	99.9% [99.9–100]
-PPV (95% CI)	15.1% [7.2–22.8]	37.1% [19.7–54.5]	26.9% [10.5–31.8]	39.0% [21.5–56.2]
-NPV (95% CI)	100% [99.9–100]	100% [99.9–100]	100% [99.9–100]	100% [99.9–100]

CI: confidence interval. PPV: positive predictive value. NPV: negative predictive value.

## Abbreviations

BW	birth weight
CI	confidence interval
CF	cystic fibrosis
CFSPID	cystic fibrosis screen positive with an inconclusive diagnosis
*CFTR*	cystic fibrosis transmembrane conductance regulator (gene)
DBS	dried blood spot
DNA	deoxyribonucleic acid
EGA	extended gene analysis
GA	gestational age
IRT	immunoreactive trypsinogen
NBS	newborn screening
NPV	negative predictive value
PAP	pancreatitis-associated protein
PPV	positive predictive value
SCT	sweat chloride test
SN	safety net
